# The Relationship Between Early-Stage Maladaptive Schemas and Treatment Adherence in Patients Diagnosed with Bipolar Disorder in Remission

**DOI:** 10.3390/jcm15093351

**Published:** 2026-04-28

**Authors:** Mahmut Onur Karaytuğ, Lut Tamam, Mehmet Emin Demirkol, Zeynep Namlı, Caner Yeşiloğlu, Sinem Çetin Demirtaş, Ülker Atılan Fedai, Ali Meriç Kurt

**Affiliations:** 1 Department of Psychiatry, Faculty of Medicine, Çukurova University, Adana 01330, Türkiye; 2 Faculty of Medicine, Harran University, Şsanlıurfa 63320, Türkiye

**Keywords:** bipolar disorder, early maladaptive schemas, treatment adherence, punishment schema, euthymia

## Abstract

**Background/Objectives:** Treatment non-adherence in bipolar disorder remains a major clinical challenge. Although demographic and clinical predictors have been widely studied, enduring cognitive vulnerability patterns such as early maladaptive schemas have received limited attention in relation to adherence behavior. **Methods:** This cross-sectional study included a total of 156 euthymic patients with bipolar disorder (HAM-D ≤ 7; YMRS ≤ 12) who were assessed using the Young Schema Questionnaire–Short Form 3, the Morisky Medication Adherence Scale, and a clinician-rated insight measure. Group differences across adherence levels were examined using ANOVA and chi-square tests. Ordinal logistic regression was conducted to identify independent factors associated with poorer adherence. Multicollinearity was evaluated, and proportional odds assumptions were tested. **Results:** Several schema domains differed significantly across adherence groups, with the Punishment schema demonstrating the largest effect size. In ordinal regression analysis controlling for age and insight, higher Punishment schema scores were independently associated with poorer adherence (OR = 1.14, 95% CI = 1.09–1.20, *p* < 0.001). Younger age and partial insight were also associated with lower adherence. **Conclusions:** Early maladaptive schemas—particularly punitive self-evaluative patterns—may represent cognitive correlates of treatment non-adherence in euthymic bipolar disorder. Interventions targeting self-critical schema processes may be relevant for adherence-focused strategies; however, due to the cross-sectional design, the observed relationships reflect associations only and do not allow for causal inferences.

## 1. Introduction

Bipolar disorder (BD) is a mental disorder characterized by episodes of mania and depression, chronic course, and high morbidity and mortality risk [1]. It is known that genetic predisposition, biological processes, psychosocial factors, and stressful life events play an important role in the onset of the disease [2,3]. Epidemiological data indicate that the 12-month prevalence of DSM-5 bipolar I disorder is approximately 1.5% in nationally representative samples, with no significant difference between men (1.6%) and women (1.5%). The lifetime prevalence ratio between men and women is reported to be approximately 1.1:1, suggesting a largely balanced sex distribution. The 12-month prevalence of bipolar II disorder has been estimated at around 0.8% in the United States and approximately 0.3% internationally. While bipolar I disorder shows a relatively equal distribution between sexes, findings regarding bipolar II disorder are more variable and appear to depend on sample characteristics and geographic context. Overall, there is limited evidence for substantial sex differences in bipolar disorder in the general population, although some clinical samples suggest a higher prevalence of bipolar II disorder among women [1]. This multidimensional etiological structure indicates that the treatment process for bipolar disorder cannot be limited to pharmacological interventions alone, but must also take into account individual psychological and cognitive factors [4]. 

Treatment adherence refers to the extent to which an individual complies with the medication and behavioral adjustments recommended by healthcare professionals [5]. Non-adherence to treatment is a common problem in bipolar disorder and is associated with increased relapse, more frequent hospitalizations, impaired functioning, decreased quality of life, and increased risk of suicide [6,7,8]. The literature reports treatment non-adherence rates in bipolar disorder ranging from 12% to 64% [9,10,11]. 

When examining factors affecting treatment adherence, variables such as young age, poor insight, negative attitudes toward treatment, fear of side effects, and comorbid alcohol or substance use stand out [12,13,14,15]. However, it is emphasized that these variables are often insufficient in explaining treatment non-adherence; there is insufficient focus on the deeper and more persistent cognitive structures underlying patients’ attitudes toward treatment [4,16]. Recent path-analytic evidence in bipolar disorder further supports this view, demonstrating that cognitive and psychological variables, including perceived cognitive failures and subjective well-being, may exert both direct and indirect effects on medication adherence beyond traditional clinical predictors [17]. These findings underscore the relevance of examining deeper cognitive processes when investigating adherence behaviors. In parallel, systematic research has conceptualized self-critical cognitive styles as transdiagnostic vulnerability factors associated with a broad spectrum of psychopathological processes, suggesting that persistent negative self-evaluative patterns may shape coping strategies and health-related behaviors [18]. These findings collectively underscore the relevance of examining enduring cognitive frameworks when investigating adherence behaviors in bipolar disorder.

Early maladaptive schemas (EMS) are cognitive-emotional structures that develop during childhood and adolescence, shape an individual’s core beliefs about themselves, others, and the world, and show relative consistency throughout life [19]. It has been reported that early maladaptive schemas are more prevalent in bipolar disorder compared to healthy controls and unipolar depression patients, with schemas such as abandonment, emotional deprivation, defectiveness, and pessimism being particularly prominent [20,21]. Furthermore, theoretical and clinical approaches to schema therapy in bipolar disorder have gained increasing attention in recent years [22]. A recent systematic review encompassing multiple psychiatric conditions, including mood disorders, has shown that early maladaptive schemas and schema modes are consistently linked to symptom severity and broader clinical characteristics, suggesting that these enduring cognitive-emotional patterns may play a transdiagnostic role in shaping illness expression [23].

However, our literature review did not identify any studies directly examining the relationship between early maladaptive schemas and medication adherence in patients with bipolar disorder in remission. Existing studies have mostly focused on defining schema levels; the relationship between these cognitive structures and behavioral outcomes related to treatment has not been sufficiently addressed. This study aims to fill this gap and identify patient-centered cognitive factors that may influence treatment adherence in bipolar disorder. The main hypothesis of the study is that early maladaptive schemas may negatively affect treatment adherence and that there may be significant differences in relative effect sizes across schema domains.

## 2. Materials and Methods

### 2.1. Participants

The study initially screened 165 individuals (79 women and 86 men) aged 18–65 who presented to the outpatient clinics of the Department of Psychiatry at Çukurova University Faculty of Medicine between 3 October 2022, and 13 October 2024, and were diagnosed with bipolar disorder according to DSM-5 criteria. All participants were patients routinely followed in the bipolar disorder outpatient clinic of the same institution. Diagnoses were confirmed by clinical interviews conducted by the first author. All authors are psychiatrists, and the diagnoses were also confirmed by the first author based on DSM-5 criteria [1].

**Figure 1:**
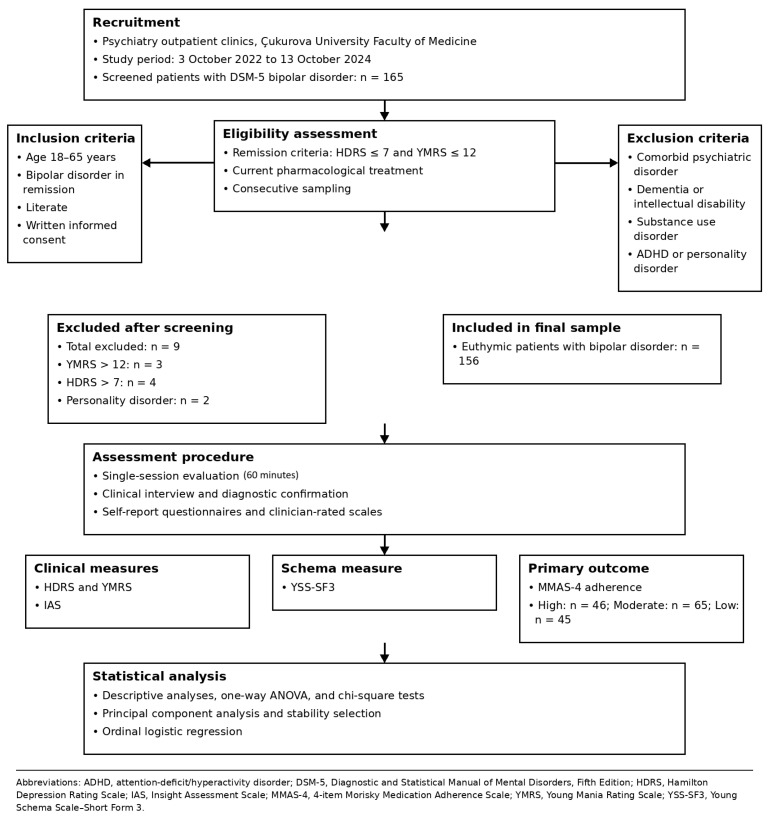
Study design and participant flow.

Inclusion criteria were being between 18 and 65 years of age, having a diagnosis of bipolar disorder in remission, currently receiving pharmacological treatment, having no comorbid psychiatric disorder, and voluntarily agreeing to participate in the study. Exclusion criteria included comorbid dementia, intellectual disability, substance use disorder, attention deficit/hyperactivity disorder, and personality disorder. The term “substance use disorder” refers to DSM-5 diagnoses. The “substance use” variable reported in the descriptive tables reflects lifetime or current psychoactive substance use and was not included as a covariate in the regression analyses. Participants were required to be literate and provide written informed consent. Consecutive sampling was used to increase representativeness. Participants were considered euthymic if they had a Hamilton Depression Rating Scale (HDRS) score ≤ 7 and a Young Mania Rating Scale (YMRS) score ≤ 12, and had not experienced any episodes or hospitalizations in the past three months. Of the 165 individuals screened, 3 exceeded the YMRS threshold (>12), 4 exceeded the HDRS threshold (>7), and 2 were diagnosed with a personality disorder; these nine individuals were excluded. The final sample consisted of 156 euthymic patients with bipolar disorder. The study design and participant flow are summarized in Figure 1. 

### 2.2. Procedure

Participants were recruited from the outpatient clinics of the Department of Psychiatry at Çukurova University Faculty of Medicine using a consecutive sampling approach. Individuals who met the inclusion criteria and did not meet any of the exclusion criteria were invited to participate in the study. After providing written informed consent, participants underwent a structured clinical evaluation conducted by the first author, during which the diagnosis of bipolar disorder was confirmed according to DSM-5 criteria and remission status was assessed. Following the clinical interview (15 min), participants completed the self-report measures (30 min), and clinician-rated scales (15 min) were administered. The entire assessment procedure was completed in a single session lasting approximately 60 min per participant.

### 2.3. Ethical Approval

Ethical approval was obtained from the Non-Interventional Clinical Research Ethics Committee of Çukurova University Faculty of Medicine (Meeting no. 115, approval date: 1 October 2021, decision no. 15). The study was conducted in accordance with the Declaration of Helsinki, and written informed consent was obtained from all of the participants.

#### 2.3.1. Variable Selection and Dimensionality Assessment

Given the high intercorrelations among schema subscales, principal component analysis (PCA) was performed to examine the dimensional structure and reduce the risk of multicollinearity. The first principal component (PC1) accounted for 46.8% of the total variance. PC1 scores were examined in alternative regression models, but the Punishment schema demonstrated superior clinical interpretability and comparable model fit and was therefore selected as the representative cognitive predictor.

#### 2.3.2. Power Analysis

A priori power analysis using G*Power 3.1 indicated that at least 119 participants were required (f^2^ = 0.15, α = 0.05, power = 0.95, three predictors). The final sample of 156 participants exceeded this requirement.

### 2.4. Measures

All participants completed a set of standardized assessment tools, and the evaluation process took 60 min per participant, including a clinical interview for diagnostic confirmation, assessment of remission status, completion of self-report questionnaires, and clinician-rated assessments.

Sociodemographic and Clinical Data Form: The data form created by the researchers included sociodemographic variables such as age (years), gender, education level, marital status, occupation, and place of residence, as well as clinical and behavioral variables including duration of illness (years), history of suicide attempts, perceived social support, and substance-related variables (smoking, alcohol use, and psychoactive substance use). Suicide attempts were defined as any action performed voluntarily with the intent to die.

Young Mania Rating Scale (YMRS): This scale consists of 11 items, each with five severity levels, and rates the core symptoms of the manic episode from mild to severe. Scoring is based on the patient’s reports and the clinician’s observations during the interview. However, the clinician’s opinion takes precedence [24]. In the Turkish validity and reliability study, the Cronbach’s alpha value was found to be 0.79 [25].

Hamilton Depression Rating Scale (HDRS): This is a 17-item measure that assesses depressive symptoms on a three- or five-point scale. High scores indicate increased severity of depressive symptoms. It is completed by the clinician. Scores of 7 or below are considered indicative of no depression [26]. In the Turkish validity and reliability study, the Cronbach’s alpha value was found to be 0.75 [27].

Young Schema Scale (YSS-SF3): Developed by Young and colleagues, the YSS-SF3 is a 6-point Likert-type measure consisting of 90 items used to assess early maladaptive schemas [19]. The scale assesses 18 early maladaptive schemas classified under five schema domains. The Turkish adaptation was conducted by Soygüt and colleagues, and the internal consistency coefficients of the Turkish form of the scale range from α = 0.63–0.80 for schema dimensions and α = 0.53–0.81 for schema domains [28]. The construct validity of the scale was supported by confirmatory factor analysis. In this study, the Cronbach’s alpha values for the scale’s subscales are: Emotional Deprivation (α = 0.72), Social Isolation/Insecurity (α = 0.82), Imperfection (α = 0.68), Suppression of Emotions (α = 0.76), Interdependence/Dependency (α = 0.83), Abandonment (α = 0.79), Vulnerability to Threats (α = 0.78), Failure (α = 0.84), Pessimism (α = 0.85), Privilege/Poor Self-Control (α = 0.83), Self-Sacrifice (α = 0.80), Punishment (α = 0.84), High Standards (α = 0.72), Approval Seeking (α = 0.83) were found.

Morisky Medication Adherence Scale (MMAS): Developed by Morisky, Green, and Levine [29]. The validity study for the scale for normal patients and its adaptation into Turkish were conducted by Yılmaz [30]. The validity and reliability study for patients with bipolar disorder was conducted by Bahar and colleagues [31]. The scale consists of 4 items. Responses to the scale are “yes” or “no,” and the patient’s medication adherence is categorized as “good,” “moderate,” or “poor” [30]. In our study, three categories were defined based on the total scores obtained from the scale: those who answered “yes” to all questions (4 points) were classified as high adherence, those who answered “yes” to two or three questions were classified as moderate adherence, and those who answered “yes” to one or fewer questions were classified as low adherence. These categories were used as dependent variables in ordinal logistic regression analysis. The Morisky Medication Adherence Scale is a widely used self-report scale in clinical research due to its short and practical structure. Although the limited number of items in the scale may constitute a limitation in terms of assessing medication adherence multidimensionally, it provides a practical assessment tool, especially in large samples and clinical settings. In this study, the ordinal structure of the Morisky scale allowed treatment adherence levels to be categorized and evaluated using ordinal logistic regression analysis. Cronbach’s α was reported to be ≈0.74.

Insight Assessment Scale (IAS): This scale consists of seven items from the Three Components of Insight Assessment Scale, translated into Turkish, with two additional items. It is a semi-structured scale administered by a clinician [32]. It has four sections that aim to assess acceptance of the illness, the ability to label psychotic experiences as abnormal, attitude toward treatment, and awareness of past mental disorders. Each item is rated on a scale of 0, 1, or 2 to measure severity. High scores indicate a high level of insight [33]. In our study, participants who scored full points on the scale were considered fully insightful, while those who did not score full points were considered partially insightful. Cronbach α = 0.778 has been reported.

### 2.5. Statistical Analysis

All statistical analyses were performed using IBM SPSS Statistics for Windows, Version 25.0, and Python 3.13.13. Continuous variables were expressed as mean ± standard deviation (SD), and categorical variables as frequency (*n*) and percentage (%). Normality was assessed using Shapiro–Wilk tests together with inspection of skewness and kurtosis values. Homogeneity of variances was evaluated using Levene’s test.

Participants were categorized into three groups (high, moderate, low) according to Morisky medication adherence levels. Group comparisons for continuous variables were conducted using one-way analysis of variance (ANOVA), and effect sizes were reported as eta-squared (η^2^). Bonferroni correction was applied to adjust for multiple comparisons.

To examine the dimensional structure of early maladaptive schema subscales and further assess multicollinearity, principal component analysis (PCA) was conducted on standardized (z-scored) schema scores. Sampling adequacy was confirmed (Kaiser–Meyer–Olkin measure = 0.873), and Bartlett’s test of sphericity was significant (χ^2^ (91) = 1264.88, *p* < 0.001), indicating suitability for factor analysis. PCA was conducted primarily as a dimensionality and multicollinearity assessment step rather than as a latent construct validation procedure. The first principal component (PC1) had an eigenvalue of 6.56 and accounted for 46.8% of the total variance, indicating a dominant general schema factor. Inspection of the scree plot showed a marked decline after the first component, suggesting a strong primary factor rather than clearly separable independent dimensions. PC1 factor loadings ranged from 0.42 to 0.83, reflecting moderate to strong contributions of all schema subscales to the general factor.

Although PC1 scores were examined in alternative regression models, the Punishment schema demonstrated superior clinical interpretability and comparable model fit. Therefore, consistent with principles of parsimony and interpretability, the Punishment schema was selected as the representative cognitive predictor in the final model.

To further address multicollinearity among schema subscales, a two-stage variable selection strategy was implemented. First, schema subscales demonstrating statistical significance after Bonferroni correction and at least medium effect size (η^2^ ≥ 0.06) were retained as candidate predictors. Second, stability selection was conducted following the approach of Meinshausen and Bühlmann, using ridge regression as the penalized base model. The ridge penalty parameter (λ) was selected via 5-fold cross-validation over a logarithmically spaced grid of candidate values and subsequently held constant across all stability selection iterations to reduce tuning variability [34].

Two hundred subsamples (50% of the sample, without replacement) were generated. Within each subsample, standardized predictors were ranked according to the absolute magnitude of their coefficients, and the top three predictors were retained. Selection probability (π) was calculated across iterations, and π ≥ 0.75 was defined as the stability threshold. Stability selection prioritizes predictors that are consistently retained across repeated subsampling procedures rather than those with large coefficients in a single penalized model. Therefore, final model selection was guided by selection probability (π) rather than coefficient magnitude alone.

Stable predictors were entered into an ordinal logistic regression model together with age and insight level. Morisky adherence level (1 = high, 2 = moderate, 3 = low) was treated as the ordinal dependent variable, with the highest adherence category serving as the reference. Regression coefficients (β), standard errors (SE), Wald z statistics, odds ratios (OR), and 95% confidence intervals (CI) were reported.

Model fit was evaluated using the likelihood ratio chi-square test and −2 Log Likelihood statistics. Pseudo R^2^ indices (McFadden and Nagelkerke) were calculated to estimate explained variance. Multicollinearity was assessed using variance inflation factor (VIF) values.

The proportional odds assumption was tested using the Test of Parallel Lines procedure. A non-significant result (*p* > 0.05) indicated that the assumption was not violated. All tests were two-tailed, and statistical significance was set at *p* < 0.05.

## 3. Results

### 3.1. Sample Characteristics and Distribution of Medication Adherence

A total of 156 patients meeting remission criteria (HAM-D ≤ 7 and YMRS ≤ 12) were included in the analyses (Table 1). The mean age of the sample was 39.6 ± 11.1 years, and 46.8% of participants were female. The mean duration of illness was 15.6 ± 9.7 years, while the mean numbers of hospitalizations, manic episodes, and depressive episodes were 3.6 ± 4.7, 4.7 ± 3.3, and 4.5 ± 3.7, respectively. Mean symptom scores were low, supporting remission status (HAM-D: 3.2 ± 1.9; YMRS: 3.6 ± 3.4). As shown in Table 1, 29.5% of participants were classified as having high medication adherence, 41.7% as moderate adherence, and 28.8% as low adherence. In addition, 41.7% of participants had full insight, whereas 58.3% had partial insight.

### 3.2. Comparison of Age and Early Maladaptive Schema Scores Across Adherence Groups

Table 2 presents comparisons of age and maladaptive schema scores across the three medication adherence groups. Age differed significantly across groups in the initial analysis (F = 3.52, *p* = 0.032, η^2^ = 0.044), with higher mean age in the high-adherence group; however, this difference did not remain significant after Bonferroni correction (*p*_adj = 0.551).

In contrast, several early maladaptive schema subdimensions showed significant differences across adherence groups. The largest effect size was observed for the Punishment schema (F = 29.54, *p* < 0.001, η^2^ = 0.279), followed by Social Isolation/Insecurity (F = 16.56, *p* < 0.001, η^2^ = 0.178), Vulnerability to Threats (F = 16.18, *p* < 0.001, η^2^ = 0.175), Privilege/Poor Self-Control (F = 14.59, *p* < 0.001, η^2^ = 0.160), and Pessimism (F = 13.06, *p* < 0.001, η^2^ = 0.146). Punishment scores increased progressively from the high-adherence group to the low-adherence group, indicating that lower adherence was associated with more pronounced punitive self-evaluative patterns. After Bonferroni correction, many schema subdimensions remained statistically significant, particularly Punishment, Social Isolation/Insecurity, Vulnerability to Threats, Pessimism, and Abandonment.

### 3.3. Stability Selection of Candidate Schema Predictors

To determine which schema variables were most robustly associated with medication adherence, the candidate schema subscales were further evaluated using stability selection based on ridge regression (Table 3). Among the included schema dimensions, only the Punishment schema met the predefined stability threshold (π ≥ 0.75), with a selection probability of 0.97 and the largest univariate effect size (η^2^ = 0.279). Although several other schema subdimensions showed moderate effect sizes in univariate comparisons, none demonstrated sufficient stability across repeated subsampling procedures. These findings supported the selection of the Punishment schema as the most stable cognitive predictor for the multivariable model.

### 3.4. Ordinal Logistic Regression Analysis of Factors Associated with Lower Adherence

The results of the ordinal logistic regression analysis are presented in Table 4. In the final model, higher Punishment schema scores were independently associated with lower medication adherence (β = 0.135, SE = 0.024, z = 5.72, *p* < 0.001, OR = 1.14, 95% CI = 1.09–1.20). Age was inversely associated with lower adherence (β = −0.038, SE = 0.015, z = −2.46, *p* = 0.014, OR = 0.96, 95% CI = 0.93–0.99), indicating that younger participants were more likely to have poorer adherence. Partial insight was also significantly associated with lower adherence compared with full insight (β = 1.332, SE = 0.343, z = 3.88, *p* < 0.001, OR = 3.79, 95% CI = 1.93–7.42). All variance inflation factor values were below 2, indicating no meaningful multicollinearity.

The overall model fit was significant (Likelihood Ratio χ^2^ = 56.27, df = 3, *p* < 0.001), and the −2 Log Likelihood decreased from 342.61 in the intercept-only model to 286.34 in the final model. The model showed moderate explanatory power (McFadden R^2^ = 0.164; Nagelkerke R^2^ = 0.287). In addition, the proportional odds assumption was not violated (Test of Parallel Lines: χ^2^ = 15.14, df = 3, *p* = 0.72), supporting the appropriateness of the ordinal logistic regression model.

### 3.5. Association Between Insight Level and Medication Adherence

Table 5 shows the relationship between insight level and medication adherence. A statistically significant association was observed between insight and Morisky adherence category (χ^2^ (2) = 17.34, *p* < 0.001), with a moderate effect size (Cramer’s V = 0.333). Partial insight was more frequent in the low-adherence group (80.0%), whereas full insight was more common in the high-adherence group (63.0%). These findings indicate that insight level is clinically relevant to treatment adherence in patients with bipolar disorder in remission.

## 4. Discussion

This study is one of the few that directly examines the relationship between early maladaptive schemas and treatment adherence in patients diagnosed with bipolar disorder in remission and addresses treatment adherence in the context of patient-centered cognitive patterns [4,10]. Our findings suggest that treatment adherence may be related not only to clinical-demographic variables but also to cognitive schema patterns that develop from the early stages [19,22]. In this respect, the study contributes to the limited literature focusing on cognitive processes in explaining treatment adherence in bipolar disorder [11,16].

Our study finding that treatment adherence increases with age is consistent with findings in the literature suggesting that older age may be associated with better medication adherence [10,35]. The higher prevalence of non-adherence in younger age groups may be related to factors such as limited experience in coping with illness, low risk perception, and more fragile attitudes toward treatment [4,14]. However, it has been reported that the age–adherence relationship is not always consistent in the literature, and that the effect of age may be weak or variable in some studies [4,36]. Therefore, it seems more accurate to consider age as a “contextual variable” within the multifactorial structure that determines treatment adherence [10,37].

Another noteworthy aspect of the present findings is that they extend the existing literature in two ways. First, previous studies in bipolar disorder have largely focused on describing the presence or severity of early maladaptive schemas, often emphasizing schemas such as abandonment, emotional deprivation, defectiveness, and pessimism in comparison with healthy controls or other clinical groups [20,21]. In contrast, the current study examined treatment adherence as a clinically meaningful behavioral outcome and demonstrated that, among multiple schema domains, the Punishment schema emerged as the most robust cognitive correlate of poorer adherence. Second, while the prior literature has consistently identified younger age and poor insight as important correlates of non-adherence in bipolar disorder [4,10,38], our findings suggest that maladaptive self-evaluative cognitive patterns may provide additional explanatory value beyond these more commonly studied clinical variables. In this sense, the present study contributes to the literature by linking schema-related cognitive vulnerability not only to symptom expression, as emphasized in previous schema research, but also to a treatment-relevant behavioral domain.

The most original finding of this study is that the punishment schema remains independently associated with treatment adherence even when controlling for age and insight. Although there are data in the literature on the presence of early maladaptive schemas in bipolar disorder, this finding is an important contribution because there are very few studies examining the direct relationship between schemas and medication adherence [20,21]. Furthermore, while schemas that are more frequently highlighted in the literature (e.g., abandonment, emotional deprivation, defectiveness) are reported at the “schema level” in most studies, in our study, a different schema (punishment) may have emerged as distinctive because we targeted “treatment adherence,” which is a behavioral outcome [4,20].

The punishment schema is characterized by the individual harshly criticizing themselves when they make a mistake, not forgiving themselves, and developing internal punitive attitudes [19,22]. The cognitive content of this schema, characterized by self-criticism and self-blame, is associated with lower treatment adherence in the present sample. For example, individuals with higher punishment schema scores may report stronger feelings of guilt and worthlessness in response to treatment-related difficulties; such emotional patterns may be associated with poorer adherence behaviors [7,14]. Indeed, it has been reported that important reasons for non-adherence include not accepting the illness, discomfort with side effects, and negative attitudes toward treatment [14,16]. From this perspective, individuals with higher punishment schema scores may also exhibit more negative beliefs and attitudes toward medication [11,16].

Selecting only a single representative schema in regression due to high inter-schema correlations strengthens the methodological consistency of the study. The fact that PCA results show that schemas largely cluster under a single dominant component suggests that placing multiple schemas in the same model may increase the risk of multicollinearity [19,21]. Therefore, selecting the punishment schema as a representative cognitive variable provided a parsimonious and clinically interpretable modeling approach. Parsimonious models are preferred both for producing more stable coefficients and for increasing clinical interpretability [4,10]. This approach may also help clarify the “critical cognitive target” that can be focused on in interventions aimed at increasing treatment adherence [11,22]. However, this approach also involves a trade-off, as focusing on a single representative schema may lead to a loss of information regarding the potential contributions of other schema domains. Given the high inter-correlations among schema domains and the dominance of a single component in the PCA results, including multiple schemas in the same model could increase the risk of multicollinearity and reduce model stability. Therefore, the selection of the Punishment schema should be interpreted as a parsimonious modeling decision rather than an indication that other schemas are not relevant. Future studies using alternative modeling approaches may further elucidate the independent and combined effects of different schema domains.

It is also important to consider that treatment adherence in bipolar disorder is a multifactorial phenomenon that may be influenced by several clinical and contextual variables beyond those included in the present regression model. Factors such as medication-related characteristics (e.g., type of medication, side effect burden, and treatment complexity), illness course variables (e.g., number of episodes, hospitalization history, and illness duration), and psychiatric comorbidities may all play a role in adherence behavior. In addition, psychosocial factors such as perceived social support, the patient–clinician relationship, and individual health beliefs may also contribute to variability in adherence. Therefore, the observed association between the Punishment schema and treatment adherence should be interpreted within this broader clinical context, taking into account the potential influence of unmeasured confounding variables.

In terms of the clinical implications of these findings, it is thought that interventions aimed at increasing treatment adherence in bipolar disorder should not be limited to pharmacological adjustments alone. Psychoeducation is one of the most studied and effective methods for increasing disease awareness, recognizing early warning signs, and strengthening treatment adherence in bipolar disorder [37,39]. Additionally, cognitive-behavioral approaches may include techniques aimed at improving adherence by targeting attitudes and beliefs about medications [11,40]. From a schema therapy perspective, punitive internal patterns such as the punishment schema may be considered relevant cognitive factors in interventions addressing treatment adherence [19,22]. Therefore, when assessing adjustment problems in bipolar patients in remission, it may be useful to screen not only for insight levels but also for punitive self-criticism patterns using clinical interviews and scales [22,33]. In this context, schema-focused psychotherapy approaches may offer more structured intervention strategies targeting punitive self-evaluative patterns. For example, techniques such as cognitive restructuring of self-critical beliefs, imagery rescripting of early maladaptive experiences, and chair work focusing on punitive internal dialogues could be adapted to address adherence-related difficulties. In addition, integrating schema-focused techniques with psychoeducation and adherence-enhancing interventions may provide a more comprehensive treatment approach. Future studies may evaluate the effectiveness of such integrated interventions in improving treatment adherence in bipolar disorder.

Patterns of self-criticism and self-blame may vary across cultural contexts, with relatively collectivistic orientations and heightened sensitivity to social evaluation potentially amplifying self-critical tendencies [41,42]. Similarly, social evaluation processes in honor, face, and reputation cultures have been linked to differences in self-perception and emotion regulation [43,44]. As an exploratory interpretation, the particularly strong association between the Punishment schema and poorer treatment adherence observed in the present Turkish sample might tentatively reflect contextual sociocultural influences related to self-evaluation, shame, and social norms. However, given that cultural variables were not directly measured in this study, this remains a speculative hypothesis that warrants direct empirical investigation in future cross-cultural research.

The present study also has several strengths. To our knowledge, it is among the first to examine the relationship between early maladaptive schemas and medication adherence specifically in euthymic patients with bipolar disorder, thereby reducing the potential confounding influence of acute mood symptoms on cognitive and behavioral assessments. In addition, remission status was operationalized using clinician-rated symptom scales, and adherence was evaluated together with insight and schema-related variables within the same analytic framework. Another strength is the use of a structured variable selection strategy combining univariate screening, dimensionality assessment, and stability selection before the final regression model, which increased the interpretability and robustness of the findings. Finally, by focusing on a clinically relevant and potentially modifiable outcome such as treatment adherence, the study provides findings with direct translational relevance for routine psychiatric care.

The cross-sectional design of the study does not allow for causal inferences. Accordingly, the observed relationships between maladaptive schemas and treatment adherence should be interpreted as correlational, and no conclusions can be drawn regarding the directionality or causal nature of these associations. Since treatment adherence was assessed using a self-report scale, there is a possibility of social desirability and recall bias [4,29]. The use of the brief MMAS-4 may have limited sensitivity in capturing the full complexity and gradations of adherence behavior. As a short self-report measure, it may be less capable of distinguishing partial, fluctuating, or context-dependent non-adherence patterns. This measurement characteristic may have influenced the strength or precision of the observed associations and should be considered when interpreting the findings. The fact that the sample consisted only of patients in remission limits the generalizability of the findings to acute episode periods [45,46]. Furthermore, the use of a single representative schema in the regression analysis limited the possibility of examining the independent contributions of other schemas and may have resulted in a partial loss of information regarding the broader schema structure. Another important limitation is that several potential confounding variables were not included in the regression model. Variables such as medication type, side effect profile, illness course characteristics (e.g., number of episodes or hospitalizations), and psychiatric comorbidities may influence treatment adherence and could partially account for the observed associations. The absence of these variables in the model limits the ability to isolate the independent contribution of schema-related factors. Since variables related to cultural interpretations were not directly measured, this section is at the level of interpretation [41,42]. No indirect indicators were systematically assessed to support this interpretation; therefore, the cultural explanation should be considered as a tentative and exploratory perspective rather than an empirically supported conclusion.

Future studies incorporating a broader range of clinical, treatment-related, and psychosocial variables may help to better control for potential confounding effects and provide a more comprehensive understanding of adherence behavior. The reliability of findings can be increased by supporting treatment adherence with objective measures (serum levels, pharmacy records, etc.) [10,36]. Furthermore, cultural context hypotheses can be tested with studies that directly measure variables such as cultural orientation, self-criticism, and guilt/shame [43,44]. Finally, randomized controlled trials evaluating the effect of schema therapy-based interventions targeting the punishment schema on treatment adherence are thought to contribute to clinical practice [11,22].

## 5. Conclusions

Treatment adherence in patients diagnosed with bipolar disorder in remission appears to be related to age, insight, and early maladaptive schemas. In particular, the punishment schema remained statistically associated with treatment adherence after adjusting for age and insight. The findings suggest that, in addition to insight and psychoeducation, targeting punitive self-criticism patterns may be beneficial in interventions aimed at improving treatment adherence.

## Figures and Tables

**Table 1:** Sociodemographic and clinical characteristics of the sample (N = 156).

Variable	Value
Age (years)	Mean ± SD = 39.6 ± 11.1
Duration of illness (years)	Mean ± SD = 15.6 ± 9.7
Gender	Female, n (%) = 73 (46.8)
	Male, n (%) = 83 (53.2)
Education level	Illiterate, n (%) = 4 (2.6)
	Primary education, n (%) = 33 (21.2)
	Secondary education, n (%) = 57 (36.5)
	University, n (%) = 62 (39.7)
Marital status	Single, n (%) = 75 (48.1)
	Married, n (%) = 81 (51.9)
Occupation	Working, n (%) = 69 (44.2)
	Not working, n (%) = 87 (55.8)
Place of residence	County seat, n (%) = 111 (71.2)
	Smaller than the provincial capital, n (%) = 45 (28.8)
Smoking	Yes, n (%) = 86 (55.1)
	No, n (%) = 70 (44.9)
Alcohol	Yes, n (%) = 34 (21.8)
	No, n (%) = 122 (78.2)
Substance use	Yes, n (%) = 4 (2.6)
	No, n (%) = 152 (97.4)
Suicide attempt	Yes, n (%) = 47 (30.1)
	No, n (%) = 109 (69.9)
Social support	Yes, n (%) = 133 (85.3)
	No, n (%) = 23 (14.7)
Insight	Complete, n (%) = 65 (41.7)
	Partial, n (%) = 91 (58.3)
Morisky adherence	High, n (%) = 46 (29.5)
	Medium, n (%) = 65 (41.7)
	Low, n (%) = 45 (28.8)

Note. Continuous variables are presented as mean ± SD; categorical variables are presented as n (%).

**Table 2:** Comparison of Age and Maladaptive Schema Scores Across Medication Adherence Groups.

Variable	High (n = 46)	Moderate (n = 65)	Low (n = 45)	F	*p*	η^2^	*p*_Adj
Age (years)	42.43 ± 9.78	39.83 ± 10.58	36.40 ±12.36	3.52	0.032	0.044	0.551
Emotional Deprivation	7.91 ± 3.72	12.09 ± 7.21	13.53 ± 7.79	9.16	<0.001	0.107	0.003
Failure	11.43 ± 6.47	15.57 ± 7.99	17.76 ± 7.16	8.82	<0.001	0.103	0.004
Pessimism	9.91 ± 5.68	12.77 ± 5.72	16.04 ± 5.78	13.06	<0.001	0.146	<0.001
Social Isolation/Insecurity	14.76 ± 8.38	19.37 ± 6.74	23.71 ± 7.34	16.56	<0.001	0.178	<0.001
Self-Sacrifice	11.50 ± 5.60	13.97 ± 5.14	16.38 ± 7.74	7.22	0.001	0.086	0.017
Abandonment	9.02 ± 4.63	11.15 ± 5.10	14.20 ± 5.60	11.77	<0.001	0.133	<0.001
Punishment	14.00 ± 6.21	18.34 ± 7.50	24.93 ± 6.38	29.54	<0.001	0.279	<0.001
Defectiveness	9.43 ± 5.43	12.34 ± 6.62	12.60 ± 5.25	4.24	0.016	0.052	0.275
Suppression of Emotions	9.93 ± 5.07	10.42 ± 4.23	14.22 ± 5.81	10.50	<0.001	0.121	<0.001
Approval Seeking	17.39 ± 7.93	21.51 ± 7.12	24.11 ± 5.95	10.53	<0.001	0.121	<0.001
Interdependence/Dependency	17.04 ± 9.88	19.69 ± 9.27	24.09 ± 8.84	6.63	0.002	0.080	0.029
Privilege/Poor Self-Control	16.96 ± 7.74	21.34 ± 6.32	24.62 ± 6.45	14.59	<0.001	0.160	<0.001
Vulnerability to Threats	9.87 ± 4.63	12.60 ± 5.07	16.02 ± 5.79	16.18	<0.001	0.175	<0.001
High Standards	8.22 ± 4.62	9.88 ± 4.41	9.91 ± 4.18	2.29	0.105	0.029	1.000

Note. One-way ANOVA was applied. Bonferroni correction was used to adjust for multiple comparisons (m = 17). Adjusted *p*-values are reported as *p*_adj (Bonferroni). η^2^ represents effect size (eta-squared).

**Table 3:** Stability Selection Results for Early Maladaptive Schema Predictors (N = 156).

Schema Subdimension	η^2^	Selection Probability (π)	Stable
Punishment	0.279	0.97	Yes
Failure	0.103	0.59	No
Interdependence/Dependency	0.080	0.46	No
Emotional Deprivation	0.107	0.42	No
Approval Seeking	0.121	0.28	No
Privilege/Poor Self-Control	0.160	0.11	No
Pessimism	0.146	<0.10	No
Social Isolation/Insecurity	0.178	<0.10	No
Self-Sacrifice	0.086	<0.10	No
Abandonment	0.133	<0.10	No
Suppression of Emotions	0.121	<0.10	No
Vulnerability to Threats	0.175	<0.10	No

Note. Stability selection was performed using ridge regression with repeated subsampling. Only schema subscales that demonstrated statistical significance after Bonferroni correction and at least medium effect size (η^2^ ≥ 0.06) in univariate analyses (Table 2) were entered into the stability selection procedure. Selection probability (π) reflects the proportion of subsamples in which a predictor was retained among the top-ranked variables. Predictors with π ≥ 0.75 were considered stable. η^2^ values represent effect sizes derived from one-way ANOVA and are presented for descriptive comparison only.

**Table 4:** Ordinal Logistic Regression Predicting Medication Adherence (N = 156).

Predictor	β	SE	Z	*p*	OR	95% CI	VIF
Punishment schema	0.135	0.024	5.72	<0.001	1.14	1.09–1.20	1.06
Age (years)	−0.038	0.015	−2.46	0.014	0.96	0.93–0.99	1.03
Partial insight (ref.: full)	1.332	0.343	3.88	<0.001	3.79	1.93–7.42	1.05

Note. β: regression coefficient; SE: standard error; Z: Wald z statistic; OR: odds ratio; CI: confidence interval; VIF: variance inflation factor. A positive β indicates an increased likelihood of lower adherence. All VIF values were <2, indicating no multicollinearity. The proportional odds assumption was met (Test of Parallel Lines, *p* > 0.05).

**Table 5:** Relationship between insight level and Morisky medication adherence (N = 156).

Insight	High Adherence	Moderate Adherence	Low Adherence
Full	29 (63.0%)	27 (41.5%)	9 (20.0%)
Partial	17 (37.0%)	38 (58.5%)	36 (80.0%)

Note. Values are presented as n (column %). Chi-square test: χ^2^ (2) = 17.34, *p* < 0.001; Cramer’s V = 0.333.

## Data Availability

The datasets generated and analyzed during the current study are not publicly available due to ethical restrictions but are available upon reasonable request and subject to ethics committee approval.

## Abbreviations

The following abbreviations are used in this manuscript:

BD	Bipolar Disorder
HDRS	Hamilton Depression Rating Scale
IAS	Insight Assessment Scale
MMAS	Morisky Medication Adherence Scale
YMRS	Young Mania Rating Scale
YSS-SF3	Young Schema Scale
